# Low internal air space in plants with crassulacean acid metabolism may be an anatomical spandrel

**DOI:** 10.1093/aob/mcad109

**Published:** 2023-08-25

**Authors:** Alistair Leverett, Anne M Borland, Emma J Inge, Samantha Hartzell

**Affiliations:** School of Life Sciences, University of Essex, Wivenhoe Campus, Essex, CO4 3SQ, UK; Department of Plant Sciences, University of Cambridge, Downing St., Cambridge, CB2 3EA, UK; School of Natural and Environmental Sciences, Newcastle University, Newcastle upon Tyne, NE1 7RU, UK; School of Life Sciences, University of Essex, Wivenhoe Campus, Essex, CO4 3SQ, UK; Department of Civil and Environmental Engineering, Portland State University, 1930 SW 124^th^ Ave., Portland, OR, USA

**Keywords:** Crassulacean acid metabolism, internal air space, leaf, mesophyll conductance, stomatal conductance, spandrel, succulence

## Abstract

Crassulacean acid metabolism (CAM) is a photosynthetic adaptation found in at least 38 plant families. Typically, the anatomy of CAM plants is characterized by large photosynthetic cells and a low percentage of leaf volume consisting of internal air space (% IAS). It has been suggested that reduced mesophyll conductance (*g*_m_) arising from low % IAS benefits CAM plants by preventing the movement of CO_2_ out of cells and ultimately minimizing leakage of CO_2_ from leaves into the atmosphere during day-time decarboxylation. Here, we propose that low % IAS does not provide any adaptive benefit to CAM plants, because stomatal closure during phase III of CAM will result in internal concentrations of CO_2_ becoming saturated, meaning low *g*_m_ will not have any meaningful impact on the flux of gases within leaves. We suggest that low % IAS is more likely an indirect consequence of maximizing the cellular volume within a leaf, to provide space for the overnight storage of malic acid during the CAM cycle.

## INTRODUCTION

**Fig. 1.**
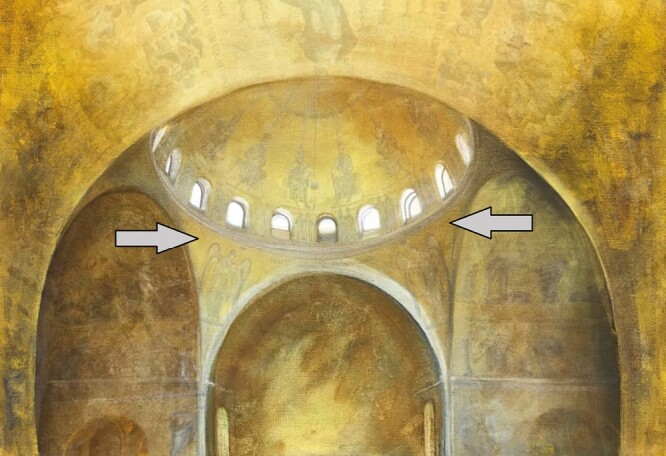
Triangular spandrels (grey arrows) are present in architectural designs when circular arches meet at right angles.

Saint Mark’s Basilica in Venice is a location of important relevance to biologists. The layout of this building was used as a metaphor by Gould and Lewontin (1979) in their seminal paper critiquing adaptationist arguments in evolution. Specifically, Gould and Lewontin discussed the spandrels inside the central dome of the cathedral: the triangular spaces that occur when circular arches meet at right angles (Fig. 1). These spandrels, they argued, may at first glance appear to be deliberate spaces left to incorporate artwork within the church. However, Gould and Lewontin further note that spandrels are just a by-product of the architectural constraints imposed when building circular, intersecting domes. Put simply, you cannot build these sorts of domes without the existence of spandrels. Gould and Lewontin proposed that an analogous concept exists in evolution, arguing that not all traits observed in nature should be considered adaptations under selection. Some traits, behaviours or even ecological interactions are simply a by-product of other traits (Raia *et al*., 2010; Freeling, 2017; Valverde *et al*., 2018). For example, the human chin is often considered a spandrel, as it does not appear to play any functional role in survival (Pampush and Daegling, 2016). Instead, this protruding bone may be a by-product of muscles and teeth shrinking during the evolutionary history of *Homo sapiens*. More recently, discussion of spandrels has been extended to the field of functional trait ecology, which aims to explain ecological patterns and distributions using physiological, biochemical and morphological traits (Violle *et al*., 2007; Volaire *et al*., 2020; Griffith *et al*., 2022). Considerable debate is ongoing about which traits should be considered ‘functional’ – i.e. which traits truly aid the growth, reproduction and survival of an organism. Some authors have suggested that all traits are functional, and that any observed trait will either interact with the environment or another trait and affect survival, reproduction or vital rates (e.g. growth) (Sobral, 2021). However, this proposal overlooks the possibility that a trait may be a spandrel: rather than contributing to survival, it is simply a by-product of another characteristic which is itself under selection (Salguero-Gómez and Laughlin, 2021).

## THE ARCHITECTURE OF PHOTOSYNTHETIC ORGANS

Most vascular plants achieve photosynthetic assimilation within leaves or photosynthetic stems. These organs contain photosynthetic parenchyma cells, whose main function is to capture light and use this energy to fix CO_2_ into sugars. Between the mesophyll cells in leaves, internal air spaces allow the diffusion and circulation of gasses. There is considerable interspecific variation in the percentage of leaf volume consisting of internal air space (% IAS) (Slaton and Smith, 2002; Earles *et al*., 2018). In plants that do typical C_3_ photosynthesis, low % IAS can limit photosynthesis as it minimizes mesophyll conductance (*g*_m_), which controls the rate at which CO_2_ can diffuse from the stomata to the chloroplasts (Baillie and Fleming, 2020; Cousins *et al*., 2020). CO_2_ diffusivity is greater within the gas than the liquid phase of cells, and hence species with a greater % IAS can conduct CO_2_ across their mesophyll more efficiently (Lawson *et al*., 2022). Additionally, the cell surface area exposed to IAS per leaf area (*L*_mes_/area) is also thought to limit *g*_m_ in C_3_ plants, by affecting the rate at which CO_2_ dissolves into the liquid phase, across the cell wall and into the chloroplast. Ultimately, reductions to *g*_m_ decrease photosynthetic assimilation rates in C_3_ leaves, as CO_2_ is slower to reach the chloroplasts (von Caemmerer, 2000).

**Fig. 2.**
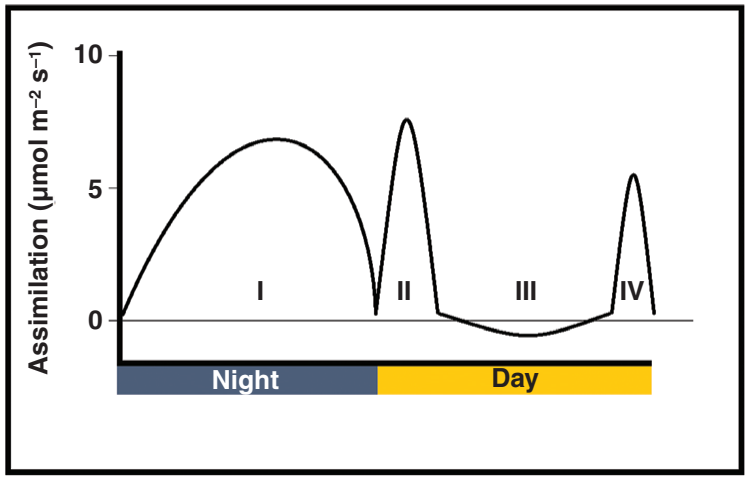
The four phases of gas exchange common to many CAM plants. Phase I: CO_2_ assimilation occurs at night, via PEPC, and carbon is stored as malic acid. Phase II: stomata open in the morning and CO_2_ is increasingly fixed by RuBisCO. Phase III: stomata shut during the middle of the day and malic acid decarboxylation produces CO_2_ to enter the Calvin–Benson–Bassham cycle. Note that during phase III some CO_2_ can leak out of imperfectly closed stomata. Phase IV: malic acid reserves are depleted, and stomata reopen; CO_2_ is fixed by RuBisCO.

Whilst the relationship between % IAS and photosynthetic assimilation in C_3_ plants is well accepted, this association is less straightforward in species that do other forms of photosynthesis (Knauer *et al*., 2022). Approximately 7 % of all vascular plants do a form of photosynthesis called crassulacean acid metabolism (CAM), which is distinct from the more common C_3_ pathway (Winter, 2019; Winter *et al*., 2021). Unlike C_3_ photosynthesis, in which CO_2_ assimilation occurs only in the day, CAM plants can exhibit four distinct phases of gas exchange over a 24-h period (Fig. 2). The majority of CO_2_ assimilation in CAM plants occurs at night (phase I), via the enzyme phosphoenolpyruvate carboxylase (PEPC). PEPC fixes CO_2_ into oxaloacetic acid, which is then converted to malic acid, transported into the vacuole and stored overnight (Borland *et al*., 2014). The following day, this malic acid is decarboxylated, thereby regenerating CO_2_ which can enter the Calvin–Benson–Bassham cycle (Ceusters *et al*., 2021). In the morning (phase II) stomata are open, allowing RuBisCO to increasingly fix CO_2_ entering the leaf from the atmosphere as well as CO_2_ generated from the decarboxylation of malic acid. However, during the hotter, drier middle of the day (phase III), CAM plants shut their stomata, and the Calvin–Benson–Bassham cycle relies entirely on malic acid decarboxylation for CO_2_ input. During phase III, some species exhibit a net efflux of CO_2_, due to imperfectly closed stomata (Friemert *et al*., 1986). Finally, in the evening (phase IV), when malic acid reserves are depleted, stomata re-open and RuBisCO fixes atmospheric CO_2_. The four phases of gas exchange in CAM plants are depicted in Fig. 2. There are two primary benefits conferred by CAM (Holtum, 2023). First, the diurnal decarboxylation of malic acid during phase III allows photosynthesis to occur without reliance on atmospheric CO_2_, meaning CAM plants can keep their stomata shut during the hotter, low-humidity portions of the day (Borland *et al*., 2015). Consequently, CAM plants conserve water, which allows them to survive in drier ecological niches (Bone *et al*., 2015; Leverett *et al*., 2021; Schweiger *et al*., 2021). The second benefit of CAM is that intercellular and chloroplastic CO_2_ concentrations (*c*_i_ and *c*_c_, respectively) are elevated during phase III, compared to ambient atmospheric conditions (Owen and Griffiths, 2013; Heyduk *et al*., 2019; Boxall *et al*., 2020). Hence, CAM is often described as a carbon-concentrating mechanism (CCM). By elevating *c*_c_ during the decarboxylation phase, CAM plants both increase the rate of RuBisCO-mediated carboxylation and decrease photorespiratory rates by reducing the wasteful oxygenation reaction also catalysed by RuBisCO (Shameer *et al*., 2018).

CAM is often found in fleshy, succulent leaves and/or stems (Borland *et al*., 2018; Males, 2018). Large succulent cells contain greater vacuole space, which aids the CAM cycle as more malic acid can be stored overnight (Töpfer *et al*., 2020; Leverett *et al*., 2023*a*). As well as large cells, another anatomical trait found in CAM plants is low % IAS (Smith and Heuer, 1981; Borland *et al*., 2018; Males, 2018). It has been proposed that unlike C_3_ photosynthesis, low % IAS actually *aids* CAM plants. Here, we critically assess the possible ways in which low % IAS could benefit CAM plants and put forward an alternative hypothesis: that this anatomical trait is best considered a spandrel, occurring as a consequence of tightly packing cells into a leaf to maximize the storage space for malic acid.

### Argument 1: Do benefits arise from low g_m_ during phase III of CAM?

The hypothesis that low % IAS is beneficial to CAM plants is based on the assumption that this anatomical configuration will lower *g*_m_, thereby slowing the rate that CO_2_ diffuses out of cells and ultimately leaks out of the leaf, during phase III. Under this scenario, low % IAS is thought to help leaves elevate *c*_c_ and enhance the CCM function of CAM. This hypothesis was first proposed by Nelson *et al*. (2005), and has been largely accepted by much of the CAM research community (Nelson and Sage, 2008; Ceusters and Borland, 2010; Ripley *et al*., 2013; Barrera-Zambrano *et al*., 2014; Heyduk *et al*., 2016*a*, *b*, 2020; Borland *et al*., 2018; Males, 2018; Edwards, 2019; Leverett, 2019; Niechayev *et al*., 2019; Gilman and Edwards, 2020; Fradera-Soler *et al*., 2021). Nelson *et al.* (2005) observed that mesophyll cell size did not correlate with % IAS across 18 phylogenetically diverse CAM species, leading them to conclude that the latter was unlikely to be an indirect consequence of developing larger cells to facilitate malic acid storage. However, a lack of any correlation between % IAS and mesophyll cell size does not necessarily mean that the low % IAS is independent of malic acid storage capacity. It is worth noting that there is no a priori reason that CAM plants need large mesophyll cells. If CAM requires adequate storage space for malic acid, this could equally be achieved through the development of a greater number of small cells. Larger cells may have simply been preferred during the evolution of CAM because it requires less carbon investment in cell walls (John *et al*., 2017). If one were to consider storage space for malic acid through the lens of total cellular volume per leaf area (*X*_vmax_), then *X*_vmax_ can be approximated by:


$$\begin{array}{l} X_{vmax}=T-\left(T\;\times \%\;IAS\right) \end{array}$$ (1)

where *T* is the thickness of mesophyll tissue, excluding tissue that does not do CAM, such as hydrenchyma (Smith *et al*., 1987; Leverett *et al*., 2023*a*). Hence, there are two ways of increasing the malic acid storage capacity for CAM: by increasing mesophyll thickness (often achieved with larger cells) and by reducing % IAS. The importance of % IAS for *X*_vmax_ can be appreciated when considering the variation across the Bromeliaceae. Earles *et al.* (2018) found that in this family, *T* was ~0.5 mm and % IAS was ~10 % in CAM species, yielding an *X*_vmax_ of 0.45. However, if CAM species had % IAS values comparable to those seen in C_3_ species (~40 %), *X*_vmax_ becomes 0.3. Hence a lower % IAS contributes to a 50 % higher *X*_vmax_ in CAM species of Bromeliaceae. This illustrates that the lack of any correlation between % IAS and mesophyll cell size does not necessarily mean that low % IAS is independent of malic acid storage capacity. Consequently, it is possible that low % IAS in CAM plants is not functionally important, but is instead a result of maximizing cell volume within leaves, to enhance malic acid storage capacity of leaves (Maxwell *et al*., 1997; Heyduk *et al*., 2016*a*, 2020; Earles *et al*., 2018).

Given that % IAS and *X*_vmax_ are often closely linked, it is difficult to compare the effect each trait has on the physiology of CAM plants. Most comparative physiology approaches would not be sufficient to determine if low % IAS aids the CAM cycle by reducing *g*_m_, or if it is a spandrel that occurs from maximizing the volume of cells. However, several models have been made that describe the physiology of CAM plants (Owen and Griffiths, 2013; Bartlett *et al*., 2014; Hartzell *et al*., 2018, 2020; Töpfer *et al*., 2020; Wang *et al*., 2023). Models can be used to artificially alter physiological and anatomical parameters, in ways that may not occur in nature. This was done by Owen and Griffiths (2013), in a model that captures all four phases of CAM. Owen and Griffiths simulated changes to *g*_m_ and *X*_vmax_, independently of one another, something that would rarely occur in natural systems. They showed that increasing *X*_vmax_ resulted in greater CO_2_ assimilation during phase I of CAM, demonstrating that greater malate storage capacity results in a direct benefit to photosynthetic assimilation rates in CAM plants. In contrast, reducing *g*_m_ had no discernible effect on CO_2_ efflux during phase III. The implication of this finding was that low % IAS (and the reduced *g*_m_ it confers) does not substantially affect *c*_i_ during phase III. Consequently, we suggest that the more likely hypothesis is that low % IAS is an indirect consequence of maximizing the cellular volume within leaves, in order to increase *X*_vmax_ and elevate nocturnal CO_2_ assimilation rates. Despite the Owen and Griffiths model having been published a decade ago, its implications for the role that low % IAS and *g*_m_ plays in CAM photosynthesis have been largely ignored.

**Fig. 3.**
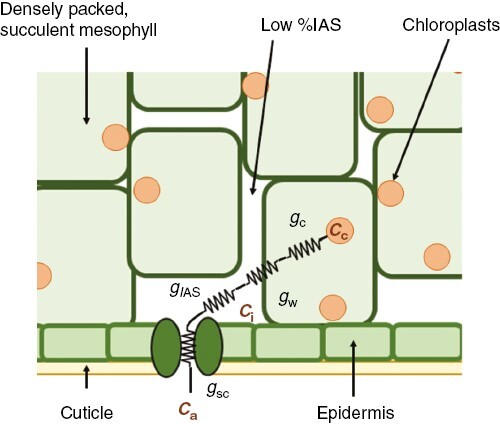
The pathway of CO_2_ diffusion away from the chloroplasts can be represented as a series of resistors with conductances: *g*_c_ (from the cell wall to the site of carboxylation), *g*_w_ (across the cell wall), *g*_IAS_ (through the intercellular air space) and *g*_sc_ (across the stomata). The mesophyll conductance, *g*_m_, can be conceptualized as comprising *g*_c_, *g*_w_ and *g*_IAS_ in series such that $g_{m}={\left(\frac{1}{g_{c}}+\frac{1}{g_{w}}+\frac{1}{g_{sc}}\right)}^{-1}$. The CO_2_ concentrations at the site of carboxylation, the intercellular air space and the atmosphere are given by *c*_c_, *c*_i_ and *c*_a_, respectively. Cell vacuoles are not shown.


We suggest that the most likely explanation for why low *g*_m_ will not substantially impact CO_2_ efflux during phase III is that stomatal resistance is far greater than mesophyll resistance during this time (note that resistance is the inverse of conductance). Low % IAS is expected to directly reduce conductance through the air space (*g*_IAS_) whilst also indirectly decreasing *L*_mes_/area, which will reduce the conductance across the cell wall (*g*_w_, see Fig. 3) (Nelson and Sage, 2008; Knauer *et al*., 2022). Both *g*_IAS_ and *g*_w_ will contribute to *g*_m_ as a whole. Thus, it is possible to predict the effect that low % IAS will have on the overall diffusion of CO_2_, by considering *g*_m_. For CO_2_ to exit a leaf during phase III of CAM, it must first travel from the cytoplasm though the mesophyll air space, then out of stomata. This can be conceptualized as two resistors in series, the first with conductance, *g*_m_ and the second with conductance *g*_sc_ (stomatal conductance to CO_2_). Therefore, the total conductance to CO_2_ between the sites of carboxylation and the leaf surface (*g*_msc_) can be derived as (Jones, 2013):

$$\begin{array}{l} g_{msc}={\left(\frac{1}{g_{m}}+\;\frac{1}{g_{sc}}\right)}^{-1} \end{array}$$ (2)

According to eqn (2), changes to *g*_m_ will have little to no impact on phase III CO_2_ losses (governed by *g*_msc_) if *g*_m_ remains substantially higher than *g*_sc_. Put simply, when *g*_m_ ≫ *g*_sc_, changes to the former will have little effect on net efflux of CO_2_ during phase III. We surveyed experimentally measured values of *g*_m_, *g*_sc_._max_ (i.e. when stomata are open) and *g*_sc_._min_ (leakiness through the cuticle and closed stomata) from the literature on CAM plants (Table 1). In *Kalanchoë daigremontiana* (Crassulaceae), *g*_sc_._min_ was three orders of magnitude smaller than *g*_m_, meaning even extremely large changes to the latter would have almost no impact on CO_2_ efflux during phase III. In *Aechmea fendleri* (Bromeliaceae), we could not find an estimate of *g*_sc_._min_ from the literature. However, the conductance of internal air space to CO_2_ (*g*_IAS_) in this species is higher than *g*_sc_._max_, which suggests that stomatal conductance is sufficiently small to prevent low % IAS from limiting CO_2_ efflux during phase III. Finally, in the C_3_-CAM intermediate species *Clusia minor*, *g*_sc_._max_ was higher than *g*_m_. However, estimates of *g*_sc_._min_ were four to six orders of magnitude lower than *g*_m_, meaning stomatal closure prohibits *g*_m_ from limiting CO_2_ efflux during phase III. Together, these data indicate that stomatal limitations to CO_2_ efflux obviate any limitations *g*_m_ can impose on the efflux of CO_2_ during phase III of CAM. The thick waxy cuticles and low stomatal densities found in most CAM species are likely to result in *g*_sc_._min_ values being orders of magnitude lower than *g*_m_ (Kerstiens, 1996; North *et al*., 2019; Kubásek *et al*., 2023). Indeed, a recent survey in the genus *Aeonium* found that conductance across closed stomata was significantly lower in CAM species than for C_3_ relatives (T. Messerschmid, personal comm.). Consequently, low % IAS is very unlikely to have any meaningful impact on *c*_i_ or the carbon balance of phase III of CAM. Taken together, these data suggest that low % IAS in CAM plants is best considered a spandrel. Future work should prioritize estimating *g*_m_, *g*_IAS_ and *g*_sc_._min_ in more constitutive CAM plants, grown under the same conditions, to understand the relative contribution each trait has on *g*_msc_.

**Table 1.** Estimates of mesophyll conductance (*g*_m_), maximal stomatal conductance to CO_2_ (*g*_sc_._max_) and minimum stomatal conductance to CO_2_ (*g*_sc_._min_), taken from the literature. Note that stomatal conductance to CO_2_ was estimated by dividing stomatal conductance to water by 1.6, to account for differences in diffusivity of H_2_O and CO_2_. The series of mesophyll and minimum stomatal conductance to CO_2_ (*g*_msc_._min_) is calculated from eqn (2).

Species	Family	*g*_m_ (mol m^−2^ s^−1^)	*g*_sc max_ (mol m^−2^ s^−1^)	*g*_sc min_ (mol m^−2^ s^−1^)	*g*_msc min_ (mol m^−2^ s^−1^)
*Kalanchoë daigremontiana*	Crassulaceae	0.053^a^	0.033^a^ or 0.023^b^	4.3 × 10^−3b^ or 0^c^	4.0 × 10^−3^ or 0
*Clusia minor*	Clusiaceae	0.1^d^	0.2^e^	4 × 10^−4f^ or 2.5 × 10^−6g^	4 × 10^−4^ or 2.5 × 10^−6^
*Aechmea fendleri*	Bromeliaceae	0.11^h,^^*^	0.007^i^	NA	NA

^a^ Maxwell *et al.* (1997). ^b^ von Caemmerer and Griffiths (2009). ^c^Friemert *et al.* (1986) – based on estimates from *Kalanchoë tubiflora*. ^d^Gillon *et al.* (1998).^e^Barrera Zambrano *et al.* (2014).^f^Leverett (2019).^g^Slot *et al*. (2021) – based on estimates from *Clusia pratensis*. ^h^Earles *et al.* (2018).^i^Males and Griffiths (2018).

^*^Value represents an estimate of internal air space conductance (*g*_IAS_), not total mesophyll conductance.

### Argument 2: Do benefits arise from low g_m_ during phase II and IV?

In addition to considerations of phase III, it has been suggested that low *g*_m_ may aid CAM by helping to trap CO_2_ during phase II, when CO_2_ entering the Calvin–Benson–Bassham cycle is derived from both the decarboxylation of malic acid and the influx through open stomata (Cousins *et al*., 2020). This hypothesis is similar to that considered in *Argument 1*: that low *g*_m_ would reduce the efflux of CO_2_ out of cells and aid CAM as a CCM. During phase II, the measured net leaf CO_2_ exchange, *A*_n_, will be determined by:


$$\begin{array}{l} A_{n}=\;V_{c}-0.5\;V_{o}-R_{d}-\;D_{c} \end{array}$$ (3)

where *V*_c_ and *V*_o_ are the carboxylation and oxygenation rates of RuBisCO, *R*_d_ is the respiratory rate and *D*_c_ is the rate at which CO_2_ is generated from the decarboxylation of malate. Thus, during phase II, a net positive *A*_n_ indicates that the rate of RuBisCO carboxylation is higher than the combined rate of CO_2_ generation from photorespiration, respiration and malate decarboxylation. Furthermore, because stomata are open during phase II, *A*_n_ can also be given by the product of the stomatal–mesophyll conductance to CO_2_, *g*_msc_, and the concentration gradient of CO_2_ from the atmosphere, *c*_*a*_, to the site of carboxylation, *c*_c_, i.e.:


$$\begin{array}{l} A_{n}=\;g_{msc}\left(c_{a}-\;c_{c}\right)=\;{\left(\frac{1}{g_{m}}+\;\frac{1}{g_{sc}}\right)}^{-1}\left(c_{a}-\;c_{c}\right), \end{array}$$ (4)

where *g*_msc_ is formulated according to eqn (2). Malate decarboxylation will elevate *c*_c_ values, although to what degree is unknown. However, because *A*_n_ is positive and *g*_msc_ is finite, *c*_c_ must be less than *c*_a_. This leads to an increase in CO_2_ uptake through the stomata, with the CO_2_ concentration in the intercellular space, *c*_i_, remaining below the atmospheric CO_2_ concentration, *c*_a_. Therefore, during phase II, a lower mesophyll conductance will decrease *g*_msc_ and should decrease net carbon assimilation. This makes sense when considering that diffusion is bidirectional. If *A*_n_ is positive, then more CO_2_ must be diffusing from the atmosphere to the chloroplasts than from the site of malate decarboxylation to the atmosphere. Changes to % IAS would have the same impact to the diffusive resistance for both fluxes. Because more CO_2_ is moving into the leaf than out, lowering *g*_m_ will result in a net reduction to *A*_n_. Consequently, whilst lower *g*_m_ will help trap CO_2_ originating from the decarboxylation of malate, this will not incur any benefit during phase II because leaf gas exchange measurements indicate inward CO_2_ diffusion is greater than leakage of decarboxylated CO_2_. Hence, the benefit of CO_2_ trapping will be outweighed by the assimilatory penalty incurred to the CO_2_ entering the leaf through open stomata. This argument also applies to other periods where *A*_n_ is positive, such as phases I and IV of CAM: whenever *A*_n_ is positive, reductions to *g*_m_ will cause CO_2_ assimilation to decrease. It is worth noting that this has additional consequences for *Argument 1*: even if low *g*_m_ does reduce CO_2_ efflux during phase III, this would only have an adaptive benefit if the CO_2_ saved were greater than the cost it would incur from reduced *A*_n_ during phases I, II and IV. Therefore, future investigations should compare closely related constitutive CAM species or populations to ask the following questions: (1) Does low % IAS result in less CO_2_ loss during phase III of CAM, and is this truly independent of *X*_vmax_? (2) Are the reductions to phase III CO_2_ efflux greater than the losses incurred to *A*_n_ during phases I, II and IV?

## IMPLICATIONS FOR CAM EVOLUTION AND CAM BIODESIGN

Low % IAS has previously been considered an important anatomical adaptation for CAM photosynthesis. However, we propose that this trait may be a spandrel: a by-product of maximizing the volume of cells within a given volume of leaf. Treating low % IAS as a spandrel will be important to the discussion on the evolution of CAM. This metabolic adaptation is thought to have evolved from C_3_ origins in at least 38 plant families, making it one of the most remarkable cases of convergent evolution in nature (Winter *et al*., 2021; Gilman *et al*., 2023). However, the intermediate steps on the evolutionary trajectory from C_3_ to CAM remain enigmatic. It has been suggested that an antagonistic relationship exists between the anatomical configuration best suited for C_3_ photosynthesis (higher % IAS, higher *g*_m_) and CAM (lower % IAS, lower *g*_m_). Such an antagonism would make it difficult to evolve a strong CAM phenotype from C_3_ origins, as it would limit the viable phenotypic space that C_3_–CAM intermediate species could inhabit (Edwards, 2019). However, if low % IAS does not confer any appreciable benefit to CAM, this antagonism would not exist. Therefore, % IAS may not constrain evolutionary trajectories across the C_3_–CAM continuum.

From a biotechnological perspective, treating low % IAS as a spandrel may also be important. Substantial efforts are underway to bioengineer the CAM pathway into C_3_ crops, to benefit from the drought tolerance this metabolic pathway confers (Borland *et al*., 2015). To achieve this goal, the anatomy of leaves in a host plant must be optimized to ensure bioengineered CAM pathways function efficiently. To this end, succulent, transgenic lines of *Arabidopsis thaliana* have been developed, which will ensure sufficient space is available for nocturnal storage of malic acid (Lim *et al*., 2018, 2020). Whilst these transgenic lines also have lower % IAS than wild-type plants, the correlation between % IAS and cell size is weak, meaning one trait can be prioritized when selecting the ideal host line for CAM biodesign (Lim *et al*., 2020). We suggest that cell size, and not % IAS, should be the criterion with which hosts are chosen, as the latter is unlikely to result in any benefits to the net carbon balance of synthetic CAM pathways. This will ensure the optimal anatomy is selected to aid with bioengineered CAM. Finally, whilst current work is attempting to express a constitutive CAM cycle in C_3_ species, future projects will probably try to bioengineer facultative CAM physiology into crops (Borland *et al*., 2018). Facultative CAM plants can switch their photosynthetic physiology from C_3_ to CAM in response to water limitations. Facultative CAM plants are therefore able to achieve higher CO_2_ uptake rates associated with C_3_ photosynthesis when growing in optimal conditions, whilst also benefiting from the water-conserving properties of CAM when water is scarce (Winter and Holtum, 2014). However, evidence from *Clusia* suggests that the anatomy of facultative CAM species often more closely resembles that of C_3_ species than constitutive CAM species (Barrera-Zambrano *et al*., 2014; Leverett *et al*., 2023*b*). The rates of C_3_ photosynthesis in well-watered facultative CAM plants are likely to be limited by *g*_m_, meaning higher % IAS would benefit facultative CAM plants that are under optimal conditions. Furthermore, having a high % IAS would confer no downside when CAM is facultatively induced, as this trait will not limit the rate of CO_2_ efflux during phase III, when the stomata are shut. In addition, higher *g*_m_ may increase the rate of photosynthetic assimilation during phases I, II and IV, as CO_2_ can more efficiently reach reaction centres. Consequently, we propose that the optimal anatomical configuration for biodesigned facultative CAM is one where % IAS is high, and *g*_m_ is comparable to values seen in C_3_ species. This would benefit plant productivity when water is available. In addition, when drought has resulted in the facultative engagement of CAM, high % IAS could result in elevated CO_2_ assimilation during phases I, II and IV, without any considerable changes to CO_2_ efflux during phase III.

## CONCLUSIONS

The anatomy of CAM plants is often associated with low % IAS, which has led some to suggest that this trait is under selection. We propose an alternative hypothesis: low % IAS is a spandrel resulting from CAM plants maximizing the volume of cells within a leaf or cladode.
